# Granzyme A-expressing Terminal Effector T Cells Dedifferentiate into Long-lived Memory T Cells

**DOI:** 10.21203/rs.3.rs-10107441/v1

**Published:** 2026-06-26

**Authors:** Nu Zhang, Chaoyu Ma, Wei Liao, Saranya Srinivasan, Chenhui he, Kenneth Fan, Tyng-An Zhou, Courtney Segura-Cepero, Heetanshi Jain, Sahana Jayakumar, Weiguo Cui, Yu Luan

**Affiliations:** University of Texas Health Science Center at San Antonio; The University of Texas Health Science Center at San Antonio; Department of Dermatology, Hunan Children’s Hospital; University of Texas Health San Antonio; University of Texas Health San Antonio; University of Texas Health San Antonio; University of Texas Health San Antonio; University of Texas Health San Antonio; University of Texas Health San Antonio; University of Texas Health San Antonio; Northwestern University Feinberg School of Medicine; University of Texas Health San Antonio

## Abstract

Memory T cells are critical for vaccines and T-cell-based therapies. However, the differentiation path of memory T cells is not completely understood. Early bulk cell population-based studies support a linear model, in which effector T cells lose the effector program and dedifferentiate into memory T cells. Recent research supports a bifurcation model, where T cells choose one of the two paths, either the effector or the memory path, at an early stage of T cell responses. Terminal effector T cells lose the capacity to become memory T cells. Here, we first demonstrate that the expression of granzyme A is largely distinct from that of granzyme B and marks terminal cytotoxic effector CD8^+^ T cells with reduced polyfunctionality. Using a granzyme A-fate mapping mouse line and an acute viral infection model, we discovered that early memory T cells are largely derived from cells lacking a history of granzyme A expression, supporting the bifurcation model. Progressively, granzyme A-expressing terminal effector T cells dedifferentiate into functional long-term memory T cells. At the late memory phase, memory T cells are a mixed population composed of cells with different differentiation paths (i.e., the linear and the bifurcated paths) that coordinate to provide balanced immune protection.

## Introduction

Memory T cells (T_MEM_) are one of the key components of immunological memory, serving as the cellular basis for various T cell-based vaccines and immunotherapies^1^. The differentiation paths of T_MEM_ have been intensively investigated. Two models, namely the linear differentiation model and the bifurcation model, have been proposed. In the linear model, all primed T cells differentiate into effector T cells. A selected subset of effector T cells can further differentiate into T_MEM_ via a dedifferentiation process (i.e., effector T cells lose the expression of effector molecules to become T_MEM_)^2–6^. In the bifurcation model, after initial priming and an early expansion phase, two subsets of T cells emerge. One will continue to acquire effector functions, including cytotoxicity. This population of effector T cells will undergo contraction after antigen clearance or become long-lived effector T cells^7^. The other subset, so-called memory precursors, will preferentially become T_MEM_ cells, including central memory T cells (T_CM_)^8–15^. It is highly possible that there are multiple divergent points contributing to the bifurcating process, including naïve T cell heterogeneity^16^, the first cell division^9, 17^, and a few cell cycles after T cell priming^12, 14^. Since the recent evidence overwhelmingly supports the bifurcation model^18^, it is generally accepted that a subset of early effector T cells maintains high Tcf-1 (T cell factor-1) expression^13^, receives less IL-2 and inflammatory signaling, has undergone less/slower cell cycles^12^, and preferentially differentiates into long-term T_MEM_, especially the T_CM_ population. Further, the memory precursors exhibit enhanced pyrimidine synthesis^15^ and are resistant to DNA damage^11^. Importantly, these memory precursor cells lack cytotoxic capacity even at the peak of responses^8^.

However, previous research is often performed using bulk T cell subsets or a snapshot of single-cell differentiation status at given stages during an immune response. Long-term tracing of terminal effector T cells in vivo is largely lacking. Whether terminal effector T cells can make a significant contribution to the long-term memory pool remains unresolved. Here, we generate a genetic fate-mapping strain of mice, in which all granzyme A-expressing effector CD8^+^ T cells are permanently labelled. We provide evidence that granzyme A expression marks terminal cytotoxic effector T cells with diminished capacity for cytokine production. Using the *Gzma*-fate mapping (*Gzma*-FM) mice, we found that the majority of T_CM_ did not experience a Gzma^+^ stage, especially at the early memory phase, consistent with the bifurcation model. However, T_CM_ that experienced a Gzma^+^ phase gradually accumulated over time to a sizable proportion. These dedifferentiated T_CM_ carried a similar level of Tcf-1 expression, exhibited subtle transcriptional changes, and elicited comparable recall responses. Thus, both linear and bifurcation models are operational following acute viral infection. The bifurcation model is the major player at the early stage of memory differentiation, whereas the linear model progressively contributes to the long-term memory T cell pool.

## Results

### Distinct expression patterns of granzyme A and granzyme B in CD8^+^ T cells.

**Figure 1:**
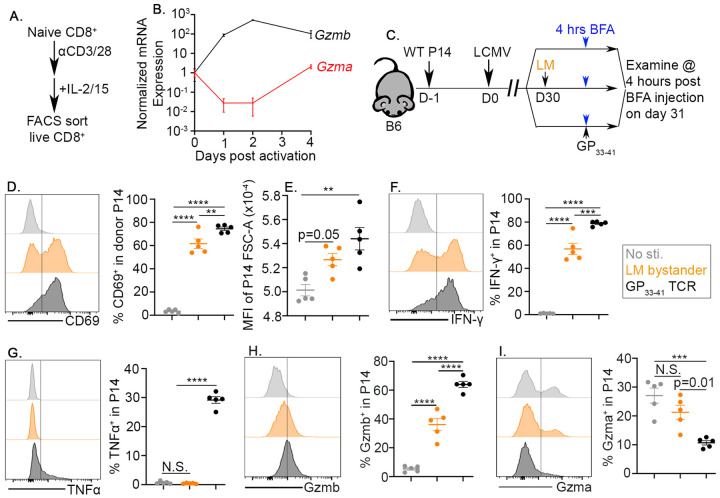
Distinct expression patterns of granzyme A and granzyme B. (**A**) Experimental setup for (B). (**B**) mRNA levels for *Gzma* and *Gzmb* during naïve CD8^+^ T cell activation *in vitro*. (**C**) Experimental setup for (D) to (I). Pre-gated on splenic P14 T cells, (**D**) the expression of CD69, (**E**) mean fluorescence intensity (MFI) of FSC-A, (**F**) IFN-g, (**G**) TNF-a, (**H**) granzyme B, and (**I**) granzyme A production are shown. Each symbol represents the results from an individual mouse. **, p<0.01, ***, p<0.001 and ****, p<0.0001 by one-way ANOVA. Representative results from two independent repeats are shown.

We first examined the expression of granzyme A and granzyme B during CD8^+^ T cell activation. Purified naïve CD8^+^ T cells were activated by aCD3/28 and cultured in IL-2/IL-15 (Fig. 1A). As shown in Fig. 1B, *Gzmb* was rapidly induced upon T cell activation. In contrast, we did not detect any upregulation of *Gzma* within the same culture period. To determine the expression of *Gzma* vs *Gzmb* in memory CD8^+^ T cells, we employed an experimental design illustrated in Fig. 1C. Briefly, naïve P14 TCR transgenic CD8^+^ T cells were adoptively transferred into C57BL/6 (B6) recipients, followed by LCMV (Lymphocytic choriomeningitis virus) Armstrong infection. P14 T cells recognize an immunodominant epitope derived from LCMV (i.e., H-2D^b-^GP_33–41_). Thirty days post-LCMV infection, memory P14 T cells were subjected to two different recall stimuli *in vivo*: antigen-specific stimulation via cognate peptide and bystander inflammation via systemic LM (*Listeria monocytogenes*) infection. To directly assess the production of effector molecules without *in vitro* culture, BFA (Brefeldin A) was injected *in vivo*. As expected, both bystander inflammation and TCR stimulation induced robust memory T cell activation as demonstrated by increased CD69 expression (Fig. 1D), cell size (Fig. 1E), and IFN-g production (Fig. 1F). Interestingly, we only detected TNF-a production in TCR, but not bystander-stimulated memory T cells (Fig. 1G). Similar to other activation markers, the expression of granzyme B was induced by both bystander and TCR stimulation (Fig. 1H). In stark contrast, the expression of granzyme A was not affected by bystander inflammation and was significantly reduced upon TCR stimulation (Fig. 1I). Thus, during the activation of both naïve and memory CD8^+^ T cells, the expression patterns of granzyme A and granzyme B are distinct.

### Generation of a *Gzma*-fate mapping (*Gzma*-FM) mouse model.

We are curious about the unique and dynamic expression pattern of granzyme A in CD8^+^ T cells. We generated a *Gzma*-Cre mouse strain by knocking in a cDNA cassette containing Cre recombinase into the endogenous *Gzma* locus. We further crossed *Gzma*-Cre mice with the *Rosa26*-LSL-tdTomato Cre reporter line to generate *Gzma*-fate mapping (*Gzma*-FM) mice (**Fig. S1A**). Presumably due to the low skipping efficiency of P2A and T2A peptides at this locus^19^, *Gzma*-Cre allele is a knockout allele for *Gzma* (**Fig. S1B** to **S1D**). We used *Gzma*-Cre/+ heterozygous mice for the following experiments to fate map *Gzma*^+^ and ex-*Gzma*^+^ cells.

In naïve *Gzma*-FM mice (*Gzma*-Cre/+; *Rosa26*-LSL-tdTomato), we detected low levels of tdTomato signal during the double negative to double positive transition of thymocyte development, which is consistent with published results that low levels of *Gzma* expression can be detected in the thymus (**Fig. S1E**)^20^. In the periphery, around 10% of both naive CD4^+^ and CD8^+^ T cells carried tdTomato expression, comparable to mature single positive thymocytes. CD44^hi^ CD8^+^, but not CD44^+^CD4^+^ T cells, contained a significantly elevated proportion of tdTomato^+^ cells. As expected, most NK cells were tdTomato^+^ (**Fig. S1F**). In the intraepithelial lymphocyte compartment of the small intestine (SI-IEL), innate-like T cells (TCRgd^+^ and CD8aa^+^TCR-b^+^) were mostly tdTomato^+^, whereas conventional TCR-b^+^ T cells, both tdTomato^+^ and tdTomato^−^ cells, were present (**Fig. S1G**). Together, we established a mouse line to fate map *Gzma* expression. Thereafter, we will term tdTomato^+^ cells in *Gzma*-FM mice as *Gzma*-FM^+^ cells.

### *Gzma*-FM faithfully marks Gzma^+^ and ex-Gzma^+^ CD8^+^ effector and memory T cells.

**Figure 2:**
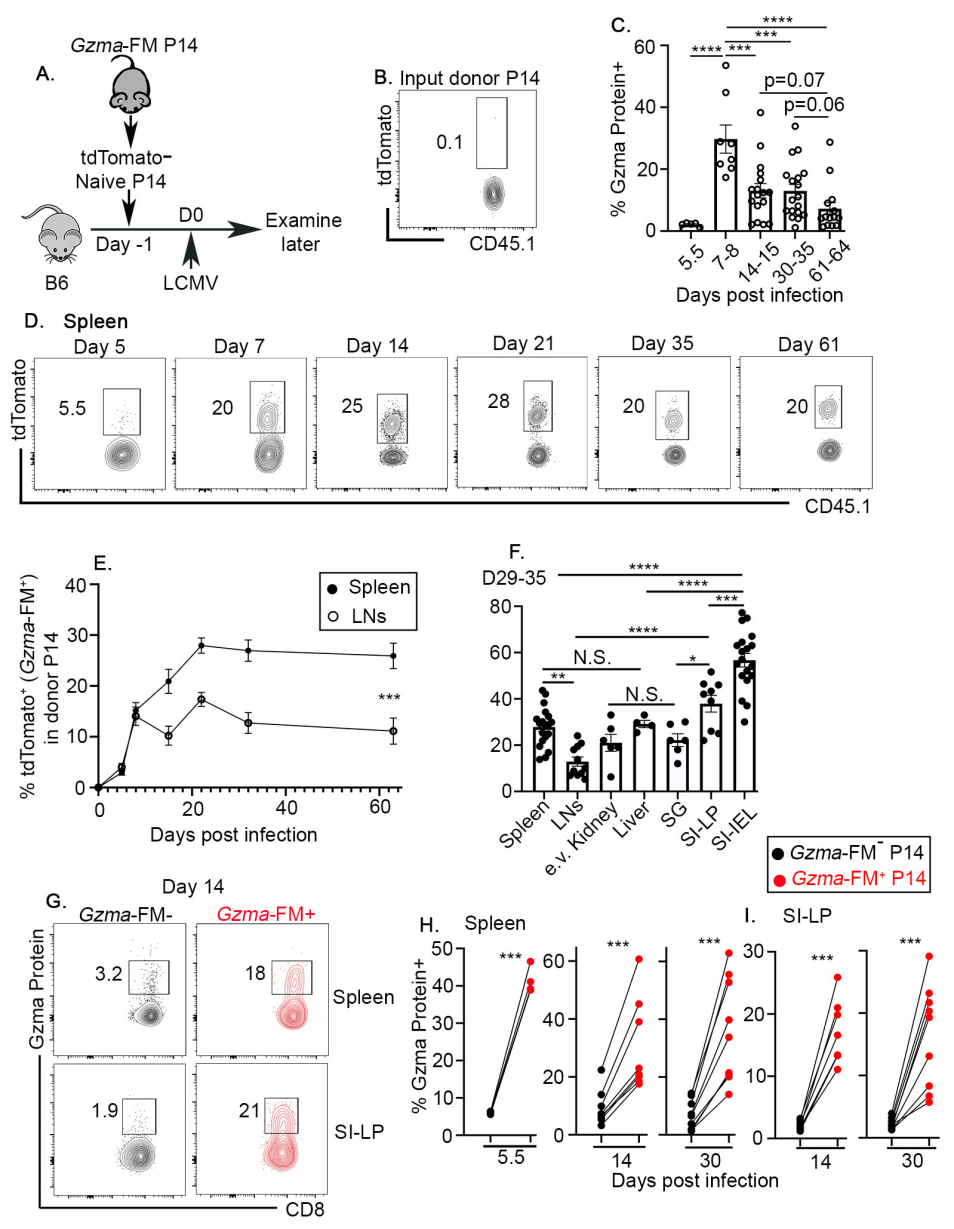
*Gzma*-FM tracks a subset of effector and memory CD8^+^ T cells. (**A**) Experimental setup. (**B**) Representative FACS profile to show input *Gzma*-FM^−^ P14 T cells. (**C**) The percentage of Gzma protein^+^ cells in donor P14 T cells isolated from the spleen is shown. (**D**) Representative FACS profiles of pre-gated donor P14 T cells isolated from the spleen. (**E**) and (**F**), the percentage of *Gzma*-FM^+^ cells within donor P14 T cells isolated from indicated tissues. Mean±SEM (n=10–20/time point) is shown in (E). (**G**) Representative FACS profiles of pre-gated *Gzma*-FM^−^ (left) and *Gzma*-FM^+^ (right) donor P14 T cells are shown. The percentage of Gzma protein^+^ cells within indicated subsets of donor P14 T cells isolated from (**H**) spleen and (**I**) small intestine lamina propria (SI-LP) is shown. Each symbol in (C and F) and each pair of symbols in (H and I) represents the results from an individual recipient mouse. *, p<0.05, **, p<0.01, ***, p<0.001 and ****, p<0.0001 by one-way ANOVA (C and F) or paired Student *t*-test (H, I). Pooled results from 4–5 independent experiments are shown.

To track antigen-specific effector and memory T cell differentiation, we backcrossed *Gzma*-FM mice onto B6 background for twelve generations and generated congenically marked *Gzma*-FM-P14 TCR transgenic mice. As shown in Fig. 2A, tdTomato^−^ naïve *Gzma*-FM P14 T cells were FACS-sorted and adoptively transferred into B6 recipients and followed by LCMV infection (input P14 purity shown in Fig. 2B). Granzyme A was rarely detectable in early effector CD8^+^ T cells (day 5.5) and reached the peak level around day 7–8 (Fig. 2C), confirming that *Gzma* expression tracks effector CD8^+^ T cell differentiation, but not T cell activation. Consistent with granzyme A expression, *Gzma*-FM signal was low at day 5 post-infection in the spleen (Fig. 2D and 2E). From the peak of P14 T cell response (day 7–8) to memory phase (>day 30–60), *Gzma*FM signal was stabilized around 20–30% of P14 T cells in the spleen (Fig. 2D to 2F). After the peak of the response, lymph node (LN) P14 T cells carried fewer *Gzma*-FM^+^ cells (Fig. 2E and 2F). Among all non-lymphoid tissues that we have examined, the small intestines harbor significantly increased *Gzma*-FM^+^ cells (Fig. 2F), consistent with the notion that intestinal mucosal tissue resident memory T cells (T_RM_) possess enhanced cytotoxicity. When comparing *Gzma*-FM^+^ vs *Gzma*-FM^−^ P14 T cells, the expression of granzyme A was largely restricted to the *Gzma*-FM^+^ subset in both spleen and small intestine, reassuring that *Gzma*-FM faithfully labels *Gzma*-expressing cells (Fig. 2G to 2I). Importantly, the frequency and distribution of *Gzma*-FM^+^ cells could be validated in a polyclonal setting in the absence of P14 TCR transgene (**Fig. S2C**).

To provide an additional control, we generated *Gzmb*-FM mice using *Gzmb*-Cre^5^ and *Rosa26*-LSL-YFP Cre reporter mice. As illustrated in **Fig. S3A**, YFP^−^ naïve P14 T cells were FACS-sorted and adoptively transferred into B6 recipients, followed by LCMV infection. Consistent with the findings that *Gzmb* is rapidly induced upon T cell activation (Fig. 1B), *Gzmb*-FM labeled more than 90% of donor P14 T cells across multiple tissues (**Fig. S3B**). Together, we conclude that *Gzma*-FM, but not *Gzmb*-FM, selectively tracks a subset of effector and memory CD8^+^ T cells starting from the peak of an immune response.

### *Gzma*-FM marks terminal effector CD8^+^ T cells without memory precursors during the effector phase.

**Figure 3:**
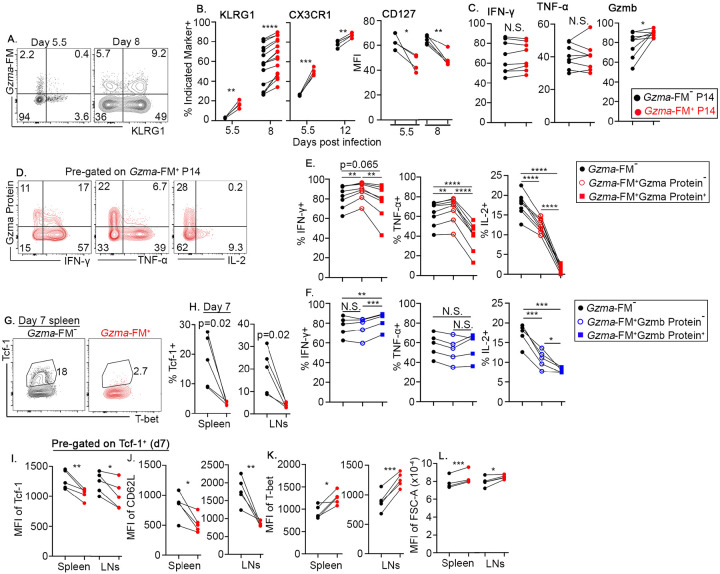
*Gzma*-FM tracks terminal effector T cell differentiation. Similar setup as in Fig. 2A. (**A**) Representative FACS profiles of pre-gated donor P14 T cells isolated from the spleen are shown. (**B**) The frequency of KLRG1^+^ (left) and CX3CR1^+^ (middle) cells, and the MFI of CD127 (right) within *Gzma*-FM^−^ and *Gzma*-FM^+^ P14 T cells are shown. (**C**) After *ex vivo* stimulation, the frequency of IFN-g^+^ (left), TNF-a^+^ (middle), and granzyme B^+^ (right) is shown. (**D**) Representative FACS profiles of pre-gated *Gzma*-FM^+^ P14 T cells (d14 spleen) are shown. (**E**) The production of IFN-g (left), TNF-a (middle), and IL-2 (right) by *Gzma*-FM^−^ (black dot), *Gzma*-FM^+^ Gzma protein^−^ (red circle), and *Gzma*-FM^+^ Gzma protein^+^ (red square) cells is shown. (**F**) The production of IFN-g (left), TNF-a (middle), and IL-2 (right) by *Gzma*-FM^−^ (black dot), *Gzma*-FM^+^ Gzmb protein^−^ (blue circle), and *Gzma*-FM^+^ Gzmb protein^+^ (blue square) cells is shown. Day 7 post-infection, (**G**) Representative FACS profiles of pre-gated *Gzma*-FM^−^ (left) and *Gzma*-FM^+^ (right) P14 T cells isolated from the spleen are shown. (**H**) The percentage of Tcf-1^+^ cells in *Gzma*-FM^−^ and *Gzma*-FM^+^ subsets is shown. Pre-gated on Tcf-1^+^ cells, (**I**) MFI of Tcf-1, (**J**) CD62L, (**K**) T-bet, and (**J**) FSC-A are shown. Each connected group of symbols represents the results from one mouse. Pooled results from 2 to 5 independent repeats are shown. N.S., not significant, *, p<0.05, **, p<0.01, ***, p<0.001 and ****, p<0.0001 by paired Student *t*-test.

To ensure that *Gzma*-FM signal is turned on specifically during effector CD8^+^ T cell differentiation, we compared *Gzma*-FM^+^ vs *Gzma*-FM^−^ circulating T cells at the effector phase of CD8^+^ T cell responses. Although *Gzma*-FM signal did not completely overlap with the expression of KLRG1 (Fig. 3A), *Gzma*-FM^+^ subset was enriched for higher KLRG1 and CX3CR1, and lower IL-7R expression (Fig. 3B), consistent with the identity of effector T cells. Importantly, *Gzma*-FM^+^ effector T cells exhibited comparable IFN-g and TNF-a production, and enhanced capacity to produce granzyme B (Fig. 3C). Thus, *Gzma*-FM signal is aligned well with an effector CD8^+^ T cell identity.

When focusing on *Gzma*-FM^+^ cells, we discovered that current granzyme A-producing cells were poor cytokine producers. Granzyme A protein^+^ cells exhibited significantly reduced capacity to produce IFN-g and TNF-a, and almost completely abolished IL-2 production (Fig. 3D and 3E). Interestingly, granzyme A protein^−^ *Gzma*-FM^+^ cells (ex-granzyme A producers) regained IFN-g and TNF-a producing capacity, even slightly and significantly higher than *Gzma*-FM^−^ counterparts. IL-2 production was also partially recovered when granzyme A production was terminated (Fig. 3E). Importantly, when dividing effector CD8^+^ T cells based on granzyme B production, we did not observe a significant reduction of IFN-g and TNF-a, while IL-2 was only slightly reduced (Fig. 3F). Thus, the expression of granzyme A labels a subset of terminal cytotoxic effector T cells, that transiently lost polyfunctionality.

Tcf-1 (T cell factor-1) is a master transcription factor and well-established marker for memory and memory-precursor T cells^21, 22^. As shown in Fig. 3G and 3H, a Tcf-1^+^T-bet^low^ subset of memory precursor cells was almost exclusively restricted to the *Gzma*-FM^−^ subset. When examining the few *Gzma*-FM^+^ cells that fell into the Tcf-1^+^ gate, these *Gzma*-FM^+^ cells carried significantly lower levels of Tcf-1 and CD62L and higher levels of T-bet compared with their *Gzma*-FM^−^ counterparts (Fig. 3I, 3J, and 3K). Further, these *Gzma*-FM^+^ “Tcf-1^+^” cells were larger in size, suggesting an activated effector, but not a quiescent memory precursor identity (Fig. 3L). This evidence strongly supports the conclusion that at the peak of CD8^+^ effector T cell responses, *Gzma*-FM^+^ subset is composed of effector T cells, lacking memory precursors. *Gzma*-FM signal is tightly linked to terminal effector CD8^+^ T cells.

### *Gzma*-FM^+^ effector T cells can dedifferentiate into central memory T cells.

**Figure 4:**
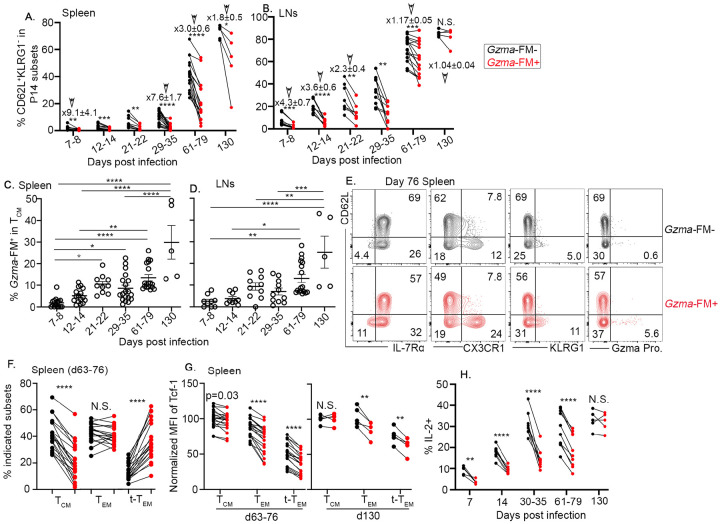
*Gzma*-FM^+^ effector T cells progressively dedifferentiate into T_CM_. Similar setup as in Fig. 2A. At the indicated times post-infection, the percentage of CD62L^+^KLRG1^−^ cells within *Gzma*-FM^−^ and *Gzma*-FM^+^ P14 subsets isolated from the spleen (**A**) and LNs (**B**) is shown. Within pre-gated CD62L^+^KLRG1^−^ P14 T cells isolated from the spleen (**C**) and LNs (**D**), the percentage of *Gzma*-FM^+^ cells is shown. (**E**) Representative flow cytometry profiles of pre-gated *Gzma*-FM^−^ (upper) and *Gzma*-FM^+^ (lower) P14 memory T cells. (**F**) The percentage of T_CM_ (left), T_EM_ (middle, CD62L^−^CD127^+^) and t-T_EM_ (right, CD62LCD127^−^) among *Gzma*-FM^−^ and *Gzma*-FM^+^ memory P14 T cells is shown. (**G**) MFI of Tcf-1 in *Gzma*-FMand *Gzma*-FM^+^ T_CM_, T_EM,_ and t-T_EM_ P14 T cells is shown. (**H**) IL-2 production from *Gzma*-FM^−^ and *Gzma*-FM^+^ P14 T cells after *ex vivo* stimulation is shown. Each pair of connected symbols in (A), (B), (F), (G), and (H), and each symbol in (C) and (D) represents the results from one mouse. Pooled results from 4–5 independent repeats. N.S., not significant, *, p<0.05, **, p<0.01, ***, p<0.001, and ****, p<0.0001 by paired Student *t*-test (A, B, F, G, H) or one-way ANOVA (C, D).

Considering the well-known effector T cell contraction process and the relatively stable frequency of *Gzma*-FM^+^ T cells (Fig. 2E), it raised the possibility that *Gzma*-FM^+^ terminal effectors can dedifferentiate into memory T cells and survive the contraction phase. To rigorously test this possibility, we examined the CD62L^+^ T_CM_ and T_CM_ precursors. At the peak of response (day 7–8), CD62L expression was essentially absent in *Gzma*-FM^+^ cells. As the response progressed into memory phase, we detected a substantial population of CD62L^+^IL-7Ra^+^KLRG1^−^ T_CM_ carrying *Gzma*-FM signal both in spleen and LNs (Fig. 4A and 4B). Although *Gzma*-FM^+^ cells carried less T_CM_ or T_CM_ precursors, the fold of reduction gradually waned. In the spleen, compared with *Gzma*-FM^−^ counterparts isolated from the same mouse, *Gzma*-FM^+^ subset carried 9.1±4.1-fold less T_CM_ precursor at day 7 to 8, 7.6±1.7-fold less at day 29 to 35, and 3.0±0.6-fold less at day 61–79 and 1.8±0.5-fold reduction at day 130. In the LNs, the values were 4.3±0.7-fold at day 7–8, 3.6±0.6-fold at day 12–14, 2.3±0.4-fold at day 21–22, 1.17±0.05-fold at day 61 to 79 and 1.04±0.04-fold at day 130 (Fig. 4A and 4B). To estimate the contribution from dedifferentiated terminal effector to memory T cells, we calculated *Gzma*-FM^+^ cell frequency within the T_CM_ population. There was a steady increase of *Gzma*-FM^+^ T_CM_ cells in both spleen and LN, reaching around 30% at day 130 post-infection (Fig. 4C and 4D). As shown in Fig. 4E, although *Gzma*-FM^+^CD62L^−^ cells were enriched for KLRG1^+^, CX3CR1^+^, Gzma protein^+^, CD127^low^ terminal effector memory T cells (t-T_EM_)^23^, *Gzma*-FM^+^CD62L^+^ cells carried comparable markers as their *Gzma*-FM^−^ counterparts. Although *Gzma*-FM^+^ memory population was composed of fewer T_CM_ and more t-T_EM_ (Fig. 4F), the expression of Tcf-1 was only slightly reduced in *Gzma*-FM^+^ T_CM_ around day 70 and completely recovered at day 130 post infection (Fig. 4G). For T_EM_ and t-T_EM_, we detected lower Tcf-1 expression in *Gzma*-FM^+^ subset (Fig. 4G). IL-2 production is a functional feature associated with memory CD8^+^ T cells, especially T_CM_. Similar to other T_CM_ markers, there was a steady increase in IL-2 production in *Gzma*-FM^+^ T cells over time. At day 130 post infection, it was indistinguishable between *Gzma*-FM^−^ and *Gzma*-FM^+^ memory T cells in IL-2 production (Fig. 4G). In addition to IL-2, *Gzma*-FM^+^ memory T cells exhibited either comparable or slightly enhanced effector cytokine production compared with *Gzma*-FM^−^ counterparts (**Fig. S5**). Furthermore, when focusing on polyclonal CD8^+^ T cells without a TCR transgene, a similar *Gzma*-FM^+^ T_CM_ subset could be identified (**Fig. S2A** and **S2B**). When enriched memory T cells (*Gzma*-FM^−^ and *Gzma*-FM^+^) were subjected to bulk RNA-seq analysis, only subtle differences were detected (**Fig. S4A** and **S4B**). These findings strongly support the conclusion that (1) although with delayed kinetics, *Gzma*-FM^+^ terminal effector T cells can dedifferentiate into T_CM_; (2) T_CM_ population is composed of two subsets with distinct differentiation history, i.e., one with a *Gzma*-expressing terminal cytotoxic stage and one without.

### *Gzma*-FM^+^ memory T cells elicit a robust recall response, comparable to *Gzma*-FM^−^ counterparts.

**Figure 5:**
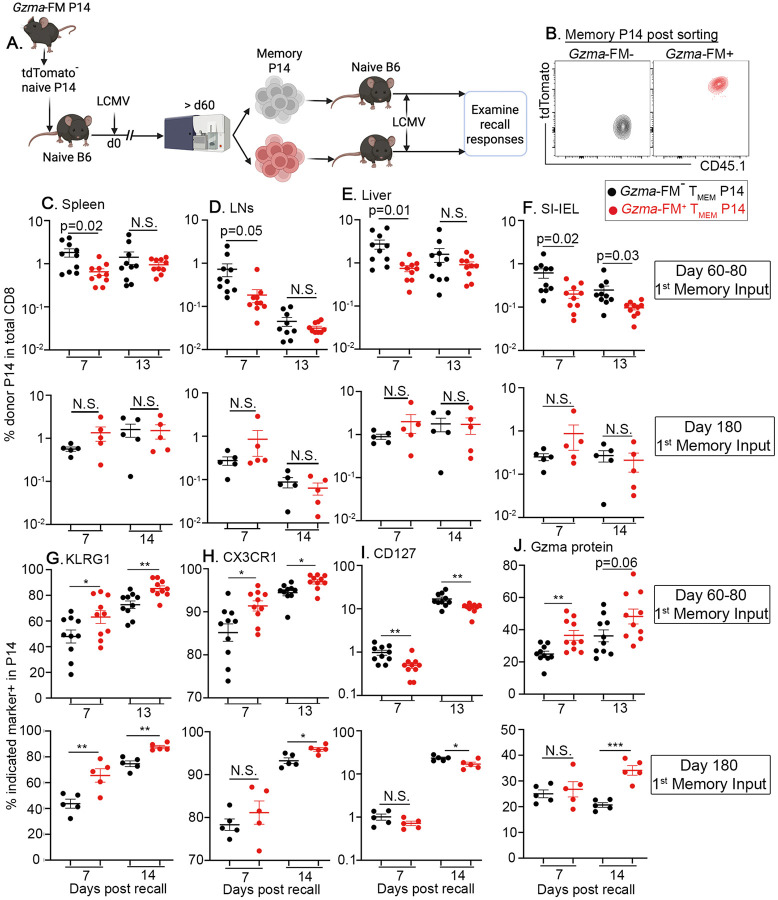
Comparable recall responses of long-lived *Gzma*-FM^+^ memory T cells. (**A**) Experimental design. (**B**) Representative FACS profiles of sorted *Gzma*-FM^−^ and *Gzma*-FM^+^ memory P14 T cells before secondary transfer are shown. At indicated times (d7 or d13–14) post recall, the percentage of donor P14 T cells in total CD8^+^ T cells in (**C**) Spleen, (**D**) LNs, (**E**) Liver, and (**F**) SI-IEL of secondary recipients is shown. The expression of (**G**) KLRG1, (**H**) CX3CR1, (**I**) CD127, and (**J**) Gzma protein in 2^nd^ effector T cells isolated from the spleen is shown. Input memory T cells were sorted from day 60–80 (upper row) and day 180 (lower row) post-primary infection. Each symbol represents an individual 2^nd^ recipient mouse. Pooled results from 3 independent repeats are shown. N.S., not significant, *, p<0.05, **, p<0.01, ***, p<0.001 and ****, p<0.0001 by Student *t*-test.

To further assess the *in vivo* recall responses of dedifferentiated memory T cells, we FACS-sorted *Gzma*-FM^+^ and *Gzma*-FM^−^ memory P14 T cells more than 60 days after the primary infection. An equal number of sorted cells were adoptively transferred into naïve secondary recipients, followed by LCMV rechallenge (Fig. 5A). When input memory T cells were isolated from d60–80 post-primary infection, *Gzma*-FM^+^ memory P14 T cells exhibited a slight but significant reduction in recall expansion at day 7. This defect was not due to altered migration because it is consistent across both lymphoid and non-lymphoid tissues (Fig. 5C to 5F top row). At day 13 post recall, the difference in the population size of 2^nd^ effector T cells had largely waned (Fig. 5C to 5F top row). As shown in Fig. 4, *Gzma*-FM^+^ effector T cell dedifferentiation is a slow and progressive process. We repeated this recall experiment using memory P14 T cells isolated from mice that received primary infection 180 days prior. In this setting, we did not detect any defects in memory T cell recall expansion (Fig. 5C to 5F bottom row). Interestingly, under both settings, we often observed significantly enhanced 2^nd^ effector T cell differentiation, including increased KLRG1, CX3CR1, granzyme A protein, and reduced IL-7R expression in *Gzma*-FM^+^ memory T cells (Fig. 5G to 5J), suggesting robust imprinting of primary effector differentiation. Together, *Gzma*-FM^+^ effector-derived memory T cells can gradually regain robust recall capacity and function as long-lived memory T cells.

## Discussion

Although it is generally considered redundant for cytotoxic T cells and NK cells, we have demonstrated that the expression of granzyme A, but not granzyme B, tracks the differentiation of terminal effector T cells with skewed cytotoxic capacity and diminished polyfunctionality. Using the *Gzma*-FM system, we discovered that the majority of circulating memory T cells, especially T_CM,_ are derived from early effector T cells without a history of granzyme A expression, supporting the bifurcation model of effector and memory T cell differentiation. Following acute viral infection, granzyme A^+^ terminal effector T cells do not represent a fixed lineage, but rather a differentiation state. When the level of granzyme A enzyme is reduced, effector CD8^+^ T cells can rapidly regain the capacity to produce cytokines. *Gzma*-FM^+^ effector T cells can dedifferentiate into functional T_CM_ cells slowly and progressively, suggesting that the linear differentiation model also contributes to the pool of memory T cells, especially at the late memory stage. Recall response is the defining characteristic of memory T cells. We provided evidence that long-term *Gzma*-FM^+^ memory T cells exhibit comparable population expansion and distribution during recall response. Interestingly, 2^nd^ effector T cells derived from *Gzma*-FM^+^ memory T cells are skewed towards KLRG1^+^ and CX3CR1^+^ effectors. This finding has two implications. First, the primary response likely leaves epigenetic marks in *Gzma*-FM^+^ memory T cells, which subsequently steer secondary effector differentiation. Second, *Gzma*-FM^−^ and *Gzma*-FM^+^ memory T cells are not completely redundant, but rather play complementary roles during memory recall responses.

As shown in Figure 1, we consistently observe reduced granzyme A expression after TCR stimulation in effector and memory CD8^+^ T cells. This striking finding can be explained by the fact that granzyme A is pre-made and stored in the mature Golgi apparatus at steady states. Upon TCR stimulation, granzyme A will be rapidly released, the process of which will not be inhibited by commonly available GolgiSTOP or GolgiPlug for intracellular flow cytometry analysis^24^. In contrast, the production of effector cytokines and granzyme B will likely initiate from mRNA translation in memory T cells and require protein export from the endoplasmic reticulum to the Golgi apparatus. Upon Golgi inhibition, cytokines and granzyme B will be accumulated inside the cells.

Previous research has demonstrated that KLRG1^+^ effector T cells can dedifferentiate into memory T cells^25^. After 30 days post primary infection, exKLRG1 memory T cells exhibited a slight but significant reduction in recall expansion. Our results show that terminal Gzma^+^ effector T cells undergo a slow dedifferentiation process and gradually regain the recall capacity.

Together, our findings demonstrate that both bifurcation and linear differentiation models are operational during memory T cell differentiation. Memory T cells are composed of a heterogeneous population of cells with distinct differentiation histories. Early bifurcated memory precursors dominate memory T cells immediately after pathogen clearance. A slowly emerging population of dedifferentiated memory T cells gradually joins the pool of circulating memory T cells to provide balanced immune protection.

## Materials and Methods

### Mice and Virus.

A colony of D^b^-GP_33–41_ TCR transgenic (P14) mice was maintained in our specific pathogen-free animal facilities at the UT San Antonio Health Science campus (San Antonio, Texas). *Gzma*-Cre mice were generated by the Mouse Genome Engineering and Transgenic Facility at UT Health San Antonio via CRISPR/Cas9-mediated homologous recombination. Briefly, a Cre-T2A-BFP-P2A cassette was inserted before the first exon of *Gzma* gene. Corrected targeting was confirmed by sequencing. Presumably due to dramatically reduced translation efficiency during T2A/P2A-mediated skipping, this is a functional knockout allele without detectable BFP signal (not shown). All experiments were performed using *Gzma*-Cre/+ heterozygous mice. C57BL/6J (B6) (Jax#000664) mice were originally obtained from the Jackson Laboratory. All recipient mice were used at 6 to 10 wk of age. All mice were housed at our specific pathogen-free animal facilities at the University of Texas Health San Antonio. All experiments were conducted in accordance with the National Institutes of Health Guide for the Care and Use of Laboratory Animals and were fully approved by the Institutional Animal Care and Use Committee of the University of Texas Health San Antonio. LCMV Armstrong infection was performed as described before^26^. 2×10^5^ pfu LCMV Armstrong was injected into each mouse via an intraperitoneal route.

### Staining for Flow Cytometry and Antibodies.

Anti-CD16/32 (2.4G2) was produced in the lab and used in all FACS staining as an FcR blocker. Freshly isolated lymphocytes were stained for surface antigens and incubated for 30 min at 4°C with the following fluorescence dye-labeled antibodies specific for CD8β (H35–17.2), CD45.1 (A20), CD45.2 (104), CD8a (53–6.7), CX3CR1 (SA011F11), CD62L(MEL-14), CD69 (H1.2F3), CD127 (A7R34), KLRG1 (2F1/KLRG1) were purchased from Thermo Fisher, Biolegend, and Cytek (Cytek/Tonbo). For live cell staining, Ghost Dye Violet 510 (Cytek/Tonbo) was used. For intranuclear staining, cells were fixed following surface staining and permeabilized with the FOXP3/Transcription Factor staining buffer kit (Cytek/Tonbo) for 45 min at room temperature and then stained with anti-Tcf-1 (C63D9, Cell Signaling) and anti-T-bet (4B10, Invitrogen). For intracellular cytokine staining, freshly isolated lymphocytes were cultured with or without 1mM GP_33–41_ peptide (AnaSpec) in the presence of Golgi Stop^™^ (BD) for 4–5 hours at 37°C. After surface staining, fixation and permeabilization using permeabilization buffer (Invitrogen), the following antibodies IL-2 (JES6–5H4), TNFa (MP6-XT22), IFNg (XMG1.2), Granzyme A (3G8.5), and Granzyme B (GB11) were incubated with the samples at 4°C for 30 min. Washed and fixed samples were collected by BD FACSCelesta, BD FACSymphony A5, and analyzed by FlowJo (TreeStar) software.

### Naïve T Cell Isolation, Adoptive Transfer and Memory Recall Response.

Naïve CD8^+^ T cells were isolated from pooled spleen and lymph nodes using MojoSort^™^ mouse CD8 T cell isolation kit (Biolegend) following the manufacturer’s instructions. Following enrichment, CD44^low^tdTomato^−^ naïve CD8^+^ T cells were FACS sorted using a BD Aria Fusion. 5×10^3^-1×10^4^ sorted cells were adoptively transferred into each sex-matched unmanipulated B6 recipient via an i.v. route followed by LCMV Arm infection.

At indicated times post-infection, lymphocytes were isolated from pooled spleen and LNs. Following CD8^+^ T cell magnetic enrichment, CD8^+^CD45.1^+^tdTomato^+^ and CD8^+^CD45.1^+^ tdTomato^−^ memory T cells were FACS sorted using a BD Aria Fusion. 5,000 to 7,000 sorted memory T cells were adoptively transferred into each sex-matched naïve B6 recipient via an i.v. route followed by LCMV Arm infection.

### Lymphocyte Isolation from Non-lymphoid Tissues.

Small intestine: Lymphocyte isolation procedures have been described before^26^. Briefly, small pieces of the small intestine were stirred at 800 rpm for 20 min in HBSS buffer containing 1mM dithiothreitol and 10% FCS at 37°C to release IEL. The remaining pieces of small intestine were further digested by 0.08U/ml Liberase TL (Sigma, 5401020001) + 200U/ml DNase I (Sigma, D5025) + 1.33mg/ml Dispase II (Sigma, D4693) with stirring for 45 min at 37°C. Both digested LP and released IEL were further purified by density gradient centrifugation with PBS-balanced 44% and 67% Percoll (GE Healthcare). Lymphocyte isolation from other non-lymphoid tissues has been described before^27, 28^. Briefly, kidney, liver, and SG was minced and digested with 1mg/ml collagenase B (Roche) in RPMI 1640/3% FCS at 37°C for 30–45 mins with gentle shaking. Digested tissues were further mashed and washed with RPMI 1640/10% FCS. Digested tissues were further purified by gradient centrifugation with PBS-balanced 44% and 67% Percoll (GE Healthcare).

### Immediate Memory Recall by Cognate Peptide or Bystander Inflammation.

After 30 days of LCMV infection, we re-challenged one group of mice with 2×10^5^ cfu/mouse LM via an i.v. route. On the following day, 250mg Brefeldin A (B6542, Sigma) in 200ml PBS was injected i.v. 4 hours before euthanasia, similar to a previous report^29^. One group of mice received 32mg/mouse GP_33–41_ peptide i.v. (Genscript) together with Brefeldin A. During the lymphocyte isolation procedure, 5mg/ml Brefeldin A was added. Freshly isolated lymphocytes were surface-stained, fixed, permeabilized, and intracellularly stained.

### Intra-vascular Labeling of CD8^+^ T Cells.

3mg biotin-aCD8a (53–6.7, Tonbo Biosciences) was injected i.v. 5 mins before euthanasia. After lymphocyte isolation, fluorescence-labeled streptavidin (Thermo Fisher) was used during surface staining to identify blood-borne CD8^+^ T cells.

### RNA-seq Analysis.

Indicated P14 T cell subsets were FACS sorted, and RNA was extracted from sorted cells using the Quick-RNA MiniPrep according to the manufacturer’s instructions (Zymo Research). RNA-seq analysis was performed by Novogene.

### Statistical Analysis.

*P* value was calculated by two-tail paired or unpaired Student *t*-test, One-way ANOVA using Prism 10 software.

## Supplementary Material

Supplementary Files

This is a list of supplementary files associated with this preprint. Click to download.

- SupplementalFiguresupdated.docx

## Data Availability

RNA-seq results can be accessed by GSE336218. Other data are available from the corresponding authors upon reasonable request.
